# Psoriasis and Psoriatic Arthritis—Associated Genes, Cytokines, and Human Leukocyte Antigens

**DOI:** 10.3390/medicina60050815

**Published:** 2024-05-16

**Authors:** Marek Zalesak, Lubos Danisovic, Stefan Harsanyi

**Affiliations:** 1 Institute of Medical Biology, Genetics and Clinical Genetics, Faculty of Medicine, Comenius University in Bratislava, Sasinkova 4, 811 08 Bratislava, Slovakialubos.danisovic@fmed.uniba.sk (L.D.); 2 National Institute of Rheumatic Diseases, Nábrežie Ivana Krasku 4, 921 12 Piestany, Slovakia

**Keywords:** psoriasis, psoriatic arthritis, HLA genetic markers, non-HLA genetic markers, cytokines

## Abstract

In recent years, research has intensified in exploring the genetic basis of psoriasis (PsO) and psoriatic arthritis (PsA). Genome-wide association studies (GWASs), including tools like ImmunoChip, have significantly deepened our understanding of disease mechanisms by pinpointing risk-associated genetic loci. These efforts have elucidated biological pathways involved in PsO pathogenesis, particularly those related to the innate immune system, antigen presentation, and adaptive immune responses. Specific genetic loci, such as TRAF3IP2, REL, and FBXL19, have been identified as having a significant impact on disease development. Interestingly, different genetic variants at the same locus can predispose individuals to either PsO or PsA (e.g., IL23R and deletion of LCE3B and LCE3C), with some variants being uniquely linked to PsA (like HLA B27 on chromosome 6). This article aims to summarize known and new data on the genetics of PsO and PsA, their associated genes, and the involvement of the HLA system and cytokines.

## 1. Introduction

Psoriasis (PsO) is a chronic systemic autoimmune-mediated inflammatory skin disease associated with many comorbidities [1]. The range includes cardiovascular diseases and metabolic syndrome, and psychological issues such as depression and anxiety frequently accompany it. The etiology of PsO remains elusive, which makes it a subject of extensive research. It is widely regarded as a multifactorial pathology, influenced by a complex interplay of immunological, environmental, and genetic factors. The relationship between psoriasis and psoriatic arthritis (PsA) is particularly significant; according to Alinaghi et al., 19.7% of patients suffering from PsO also experience PsA, which is characterized as an inflammatory arthritis occurring in conjunction with psoriasis [2]. The co-occurrence of these conditions often complicates diagnosis and management, underscoring the need for integrated therapeutic strategies.

In 2016, the World Health Organization (WHO) addressed the challenges of psoriasis globally in its *Global Report on Psoriasis* [3]. The report highlighted a considerable gap in robust data concerning the incidence and prevalence of PsO. This lack of data poses challenges in comparing studies due to varied methodologies and sampling techniques. Prevalence estimates for PsO, based on a review of 68 articles from 20 countries, range dramatically, ranging from as low as 0.09% to as high as 11.4% [4,5]. Similarly, an extensive Italian study spanning from 2001 to 2005 observed PsO incidences of between 2.3 and 3.21 cases per 1000 individuals [6]. Genetic factors play a crucial role in both PsO and PsA, substantiated by familial aggregation, twin studies, and broad population-based studies [7,8]. Notably, the concordance rates of monozygotic twins range between 35% and 70%, while for dizygotic twins, the rates are between 12% and 23% [9]. These studies suggest a strong genetic component in the pathogenesis of PsO. Recent advances in genetic research, including linkage studies, genome-wide association studies (GWASs), and genome-wide meta-analyses, have been pivotal in identifying over 80 genes and loci that contribute to PsO susceptibility [10]. These discoveries not only enhance our understanding of the genetic architecture of PsO, but also pave the way for future genetic-based therapies and personalized medicine approaches.

This article aims to summarize known and new data on the genetics of PsO and PsA, their associated genes, and the involvement of the HLA system and cytokines.

## 2. Genetic and Pathogenetic Aspects of Psoriasis

The pathogenesis of PsO is complex, involving not only extensive interactions between immune cells and skin structures but also a strong genetic component that underlies its development and progression [11]. In PsO, keratinocytes proliferate abnormally fast, which is thought to be a response to chronic inflammatory signals. These signals originate from a variety of immune cells that infiltrate the skin, including T-cells, macrophages, and dendritic cells, contributing to the characteristic thick, scaly skin lesions seen in psoriasis.

Research has identified T-cells, particularly those expressing Th1 and Th17 cytokines, as key players in the maintenance of psoriatic plaques. These T-cells help drive the inflammatory process through the release of cytokines such as IFN-γ, IL-17, and TNF-α, which not only promote the proliferation of keratinocytes but also enhance the infiltration of additional immune cells into the skin [12,13]. Furthermore, the role of the immune system in PsO extends beyond adaptive immunity. The innate immune system, which includes natural killer cells, macrophages, and neutrophils, also plays a crucial role in the initiation and perpetuation of the disease. For instance, research has shown that psoriatic lesions contain an increased number of Langerhans cells and other antigen-presenting cells, which may contribute to the activation and maintenance of T-cell responses in the skin [14].

At the genetic level, PsO and psoriatic arthritis (PsA) share a common genetic background, with the major genetic risk factor localized to the MHC class I region on chromosome 6p21.3. This region encodes for several immune-related genes, contributing significantly to genetic susceptibility to psoriatic disease. Key genes in this region include those related to the HLA complex, which was first linked to PsO in the early 1970s, illustrating the long-standing recognition of genetic factors in PsO pathogenesis [15]. Recent advances in genetic research, particularly genome-wide association studies (GWASs), have identified numerous single-nucleotide polymorphisms (SNPs) associated with PsO. These genetic variants frequently occur in genes involved in immune regulation, such as those encoding cytokines and their receptors. For example, variants in IL-23R, which encodes the receptor for IL-23, are associated with PsO. IL-23 is critical for the differentiation and maintenance of Th17 cells, a T-cell subset strongly implicated in psoriasis due to its production of IL-17 [16]. Environmental factors also play a significant role in triggering or exacerbating PsO, especially in individuals with a genetic predisposition [17]. These factors include infections such as streptococcal pharyngitis, physical trauma to the skin (Koebner phenomenon), smoking, obesity, and significant psychological stress, while obesity is often named the main risk factor for developing psoriatic disease and it also increases the likelihood of PsA [1]. Each of these factors can initiate or worsen the inflammatory cycle that is characteristic of PsO, highlighting the complex interplay between genetics and the environment in the pathogenesis of psoriasis.

With these facts in mind, we can say, that PsO is a multifaceted disease characterized by both immune-mediated inflammation and significant genetic contributions. While much progress has been made in understanding the genetic underpinnings and immune pathways involved in PsO, ongoing research continues to unravel the complex genetic architecture and the myriad environmental interactions that influence the disease trajectory.

## 3. Associated Genes

The genetics of PsO reveal a rich and intricate tapestry of gene interactions and pathways, highlighting the multifactorial nature of this disease. Understanding the genetic basis of PsO has led to targeted therapies, particularly those that inhibit the Th17/IL-23 pathway [13]. Drugs targeting IL-23, such as ustekinumab, have been effective in reducing the severity of PsO by directly influencing one of the central pathways in its pathogenesis. These genetic insights not only enhance our understanding of PsO but also guide the development of more precise and effective treatments, improving outcomes for patients with this challenging condition. Genes implicated in PsO have been categorized into different functional groups, each contributing differently to the disease mechanism and therapeutic targeting:

1. Antigen Presentation: HLA-Cw6, ERAP1, ERAP2, MICA—These genes are critical for the presentation of antigenic peptides to T-cells. HLA-Cw6, for example, is one of the most strongly associated genetic markers for PsO, influencing how the immune system recognizes and responds to pathogens and self-antigens.
2. IL-12/IL-23 Axis: IL12Bp40, IL23Ap19, IL23R, JAK2, TYK2—This group of genes regulates cytokines that are pivotal for T-cell differentiation, particularly into Th1 and Th17 cells. The IL-23 receptor pathway, through its influence on Th17 cell functioning, is a primary therapeutic target, as evidenced by the efficacy of biologics that block IL-23.
3. T-cell Development and Polarization: RUNX1, RUNX3, STAT3, TAGAP, IL-4, IL-13—These genes are involved in T-cell lineage decisions and the polarization of T-cells into specific subtypes crucial for PsO pathogenesis, such as Th2 cells, which are influenced by IL-4 and IL-13.
4. Innate Immunity: CARD14, c-REL, TRAF3IP2, DDX58, IFIH1—These genes encode proteins that play roles in the innate immune response, providing the first line of defense against pathogens and initiating inflammatory responses that can lead to psoriatic plaque formation.
5. Negative Regulators of Immune Responses—TNIP1, TNFAIP3, NFKBIA, ZC3H12C, IL36RN, SOCS1: These genes help modulate and dampen the immune response, preventing uncontrolled inflammation. Dysregulation of these genes can lead to the prolonged inflammatory responses seen in PsO.

### Psoriasis Susceptibility Loci (PSORS)

Research has identified multiple chromosomal regions, known as PSORS loci, that harbor genes linked to PsO. To date, at least 12 PSORS loci have been recognized. These loci represent regions of the genome where variations can significantly increase the risk of developing PsO. Table 1 provides an overview of known loci and their associated genes.

## 4. Human Leukocyte Antigens

The HLA system plays a crucial role in the immune system’s ability to distinguish between self and non-self. This system’s association with PsO provides insight into the genetic basis of this complex autoimmune condition. The variations in HLA class I (A, B, C) and class II (DR, DQ) antigens have been extensively studied to understand their roles in PsO susceptibility and progression.

HLA Class I Associations

HLA-A: In their research findings, Singh et al. noted a significantly higher prevalence of HLA-A1, A24, and A28 subtypes in psoriatic patients compared to healthy controls. This suggests a predisposition that may enhance the presentation of pathogenic peptides to immune cells, triggering an autoimmune response [30].

HLA-B: Interestingly, in cases without arthritis, the HLA-B13 subtype was more common in healthy controls than in psoriatic patients, which might indicate a protective effect against PsO. In cases with arthritis, studies identified subtypes such as HLA-B7 and B27 as being particularly associated with PsA, pointing to a genetic link between PsO and joint inflammation. According to other authors, HLA-B13, -B16, -B17, -B38, -B39, and -Cw6 are associated with or without arthritis [31].

HLA-C: The Cw6 subtype shows a strong association with PsO, which is significantly elevated among psoriatic patients. This subtype is known to affect the immune system’s response, possibly by influencing the skin’s inflammatory environment [32,33].

HLA Class II Associations

HLA-DR3 and DR53 were found at higher rates in psoriatic patients. The presence of DR3 exclusively in psoriatic patients underlines its potential as a biomarker for PsO susceptibility. HLA-DQ1 co-occurrence with DR53 further supports the idea of a multifactorial genetic landscape influencing PsO pathology.

## 5. Cytokines

Cytokines play a pivotal role in facilitating the interactions between cells that lead to the abnormal structures and functions observed in PsO. These include the abnormal proliferation and differentiation of keratinocytes, excessive growth of blood vessels, activation of immune cells, and promotion of abnormal immune responses. Pro-inflammatory cytokines, in particular, are critical in the development and exacerbation of PsO [34,35,36]. Secukinumab, an IL-17A antibody, and Guselkumab, a selective IL-23 inhibitor, were developed and tested based on the IL-23/IL-17 axis [37,38,39]. Also, non-coding RNAs (ncRNAs) have been studied in association with connective tissue metabolism, inflammation, and cell proliferation, linking them to cytokine signaling and T-cell activation [40,41,42]. Type I Interferons (INFs) are products of plasmacytoid dendritic cells (DCs) in the early psoriatic stage. Type I IFNs modulate the production of IFN-γ and IL-17 and play a crucial role in the differentiation and activation of T-cells, especially Th1 and Th17 cells [43].

IL-12 and IL-23 are heterodimeric pleiotropic proteins that share the p40 subunit, which is encoded by IL12B, which is vital for the differentiation of Th1 and Th17 cells, respectively. IL-12 also includes a distinct p35 subunit, while IL-23 has a unique p19 subunit that is encoded by IL23A. Elevated expressions of the p19 and p40 subunits have been observed in psoriatic skin lesions, whereas the expression of the p35 subunit does not increase, highlighting the significance of IL-23 in the pathogenesis of PsO [12,44]. So, the final effect on psoriatic susceptibility depends on the IL-12/IL-23 ratio [45,46].

IL-17, the main cytokine produced by Th17 cells, plays a crucial role in the pathogenesis of psoriasis. Additionally, neutrophils, mast cells, and natural killer (NK) cells also produce IL-17, which is implicated in the pathogenesis of inflammatory bowel diseases [47]. IL-17 is considered the primary regulator of psoriatic cutaneous inflammation and is key in linking the innate and adaptive immune responses [48,49,50]. IL-17, particularly IL-17A, emerges as a significant cytokine in the pathogenesis of psoriatic disease. Mast cells, γδ T-cells, αβ T-cells, and innate lymphoid cells are the primary sources of IL-17A in the lesioned skin and synovial fluid of patients. IL-17A targets various cells, including keratinocytes, neutrophils, endothelial cells, fibroblasts, osteoclasts, chondrocytes, and osteoblasts in the skin and joints, stimulating them to produce antimicrobial peptides, chemokines, and pro-inflammatory cytokines. This activity promotes tissue inflammation and bone remodeling, highlighting the critical role of the IL-23/IL-17A axis in the disease’s pathogenesis, leading to new biologic treatments targeting these cytokines [51,52].

IL-22 stimulates keratinocytes in human skin in various ways. In conjunction with IL-17, it can induce keratinocyte proliferation and suppress their differentiation during the tissue-remodeling phase seen in PsO [53]. IL-9, a pro-inflammatory cytokine, enhances the production of IL-17, IL-13, IFN-γ, and TNF-α in psoriasis, contributing to the inflammatory response [35]. IL-33 is a recently discovered mediator of the IL-1 family [54]. Certain cytokines, like HLA antigens, are linked to risks for both psoriasis and psoriatic arthritis. Genetic associations with both conditions have been identified for cytokines such as IL-12B, IL-23A, IL-23R, IL-2/IL-21, and TNF-α [14].

Tumor necrosis factor α (TNFα) plays a significant role in various inflammatory skin conditions, including psoriasis. TNF-α influences the antigen-presenting capabilities of DCs and stimulates T-cell infiltration [55]. It also activates the expression of C-reactive protein, a component of the acute phase response, and various cytokines, such as IL-6 and IL-23, in addition to inducing chemokines like CXCL8/IL-8 and CCL20, which are crucial for neutrophil and myeloid DC and Th17 cell recruitment, respectively. TNF-α is a vital regulator of the IL-23/Th17 axis in PsO [56].

## 6. Genetic and Pathogenetic Aspects of Psoriatic Arthritis

Psoriatic arthritis (PsA) is an inflammatory arthritis that occurs in 20–30% of patients diagnosed with psoriasis [57]. PsA is influenced by genetic, immunologic, and environmental factors, with epidemiological studies indicating a strong genetic component [58,59]. The genetic predisposition to PsA is significant, as evidenced by a recurrence rate of 30–55% among siblings and first-degree relatives [60,61]. The adoption of GWASs, including the use of the immunochip, has profoundly shifted our understanding of disease pathogenesis in psoriasis PsO [62]. These studies, involving over 15,000 PsO cases and 27,000 healthy controls, have identified more than 60 risk loci and have also elucidated pathways involved in the pathogenesis of PsO, specifically those related to the innate immune system, antigen presentation, and the acquired or adaptive immune response [63,64]. Genetic research has uncovered significant dominant effects of the major histocompatibility complex (MHC) region, including both HLA and non-HLA alleles. Genome-wide association studies have played a pivotal role in pinpointing key genes within critical signaling pathways, such as IL-23/IL-17, RANK, and NFκB [65].

Given that PsA frequently occurs alongside PsO, with an estimated prevalence of 30%, it is not unexpected that many of the genetic variants identified are common to both conditions. However, GWAS scans and meta-analyses focused on PsA, involving over 3000 PsA cases and 13,000 controls, have identified fewer variants reaching genome-wide significance. Over 20 variants have achieved this threshold, including genes like HLA-A, HLA-B, HLA-C, IL-12B, IL-23R, IL-23A, and others, highlighting the genetic underpinnings specific to PsA [64,66,67,68,69]. Similar to PsO, GWAS scans and meta-analyses in PsA have revealed pathways involved in its pathogenesis, particularly those related to the innate immune system, antigen presentation, and the acquired or adaptive immune response [70,71]. These findings have identified key signaling pathways and genetic markers, such as those affecting epidermal differentiation, innate immunity, antigen processing, and adaptive immunity, that are crucial in understanding the mechanisms of PsA. HLA genetic markers specifically associated with PsA are detailed further in Table 2.

To discern PsA-specific or PsA-weighted genetic variants, researchers compared GWAS results from PsA patients to those from patients with cutaneous PsO without joint involvement. Identifying risk loci unique to the development of PsA in PsO patients has proven challenging, though emerging evidence highlights loci reaching genome-wide significance that are uniquely associated with PsA and not PsO, such as CSF2, PTPN22, TNFAIP3, HLA-B, and IL23R [64,66,76,84]. The majority of genetic findings in these studies show only modest odds ratios, except for in the HLA region. Specific alleles such as HLA-B08, HLA-B27, HLA-B38, and HLA-B39 are linked with a significantly increased risk of developing PsA. Conversely, HLA-C*06 is associated with a reduced risk of PsA, acting as a protective factor in comparison to patients with PsO. However, a recent study suggests that the genetic variance for PsA may not be as extensive as previously thought [85].

In vitro models observed the impact of genistein (a soy-derived isoflavone known for its anti-inflammatory properties) on psoriatic cells [86]. Different studies on the dietary effects of PsA focused on omega-3 and omega-6 fatty acids, reporting that certain genetic profiles might reduce the risk of PsA in the presence of a high dietary intake of omega-3 [87]. Lysosomal dysfunction in psoriasis might also contribute to chronic inflammation [88]. Lin et al. identified an abnormality in the apoptosis of CD14+ monocytes, which can lead to prolonged inflammatory responses, in patients with PsA [89]. Table 3 presents pooled data based on the latest research and data from two recent systematic reviews, showing non-HLA genetic markers categorized into three levels of evidence: strong, moderate, and conflicting [90,91]. No genetic markers reached a strong level of evidence for a definitive positive, negative, or neutral association with the presence of PsA.

## 7. Conclusions

Reviews in the field of PsO and PsA focus on evaluating key genetic markers related to the most significant HLA markers for PsO and PsA, and the IL-12–IL-23–IL-17 axis [81,91,99]. The genetic marker HLA-C06, also known as PSOR1, is particularly crucial, accounting for up to 50% of the heritability of PsO in the general population. Within PsO populations, studies show that patients with PsO who also have the HLA-C*06 marker are less likely to develop PsA [100]. Despite numerous studies on this association, further high-quality research is needed to confirm the largely negative relationship between HLA-C*06 and the onset of PsA. A recent case-control study found that the polymorphisms studied in the IL-12B and IL-23R genes did not show a significant association with psoriasis susceptibility in a southern European cohort [101].

This review found moderate evidence supporting the association of HLA-B*27 with concurrent PsA in patients with PsO. This marker is notably prevalent (90%) in ankylosing spondylitis (AS), and while it is also more common in other spondylarthritis conditions than in the healthy population, it is less frequent than in AS [102,103]. HLA-B27 levels were found to be higher in PsO patients who developed arthritis compared to those who did not. This suggests that HLA-B27 might help differentiate between PsO patients who will and will not develop PsA, considering that both conditions are part of the spondylarthritis spectrum.

From a genetic perspective of the IL-17/IL-23 axis, there was moderate evidence indicating no significant differences in SNPs in the IL23 gene between PsA and PsO patients. However, the review highlighted limited evidence showing a higher occurrence of the SNP rs79877597 in the IL17 gene in PsA patients compared to PsO patients. While the IL-17/IL-23 axis is important for the development of psoriatic disease in general, these findings suggest its limited relevance in the development of PsA among PsO patients.

Studies on the incidence of PsO and PsA in first- and second-degree relatives have indicated stronger heritability of PsA compared to psoriasis alone. This suggests the existence of PsA-specific risk loci. Identifying such loci could aid in developing therapies that are more effective for PsA, especially as a considerable portion of patients are non-responsive to current treatments. Notably, evidence of a PsA-specific locus has been found at HLA-B27 within the MHC region. Recent studies have also identified non-HLA risk loci specific to PsA at IL23R, PTPN22, and on chromosome 5q31. Further functional characterization of these loci will enhance understanding of the pathways underlying PsA and facilitate the application of genetic findings in patient therapy [34]. Diagnosing PsA presents unique challenges due to its greater clinical heterogeneity compared to other autoimmune diseases, like PsO or Rheumatoid Arthritis (RA). This diversity in symptoms and disease manifestations, coupled with a lower rate of accurate diagnoses, complicates the ability to conduct consistent and reproducible genetic research. As fewer patients are correctly diagnosed, gathering reliable data for genetic studies is hindered, impacting the development of targeted therapies and advancements in understanding the genetic foundation of PsA [104,105].

Achievements with TNF inhibitors have led to significant improvements across multiple aspects of psoriatic arthritis. These improvements encompass not only the primary signs and symptoms of arthritis but also extend to dactylitis and enthesitis, as well as skin manifestations. Furthermore, there have been enhancements in functional status and quality of life, along with a notable reduction in the progression of radiographic damage [106]. While combination therapy has been shown to be very effective for plaque psoriasis, with promising combinations of pioglitazone, the overall approach remains complicated and complex, especially for patients with serious comorbidities [107,108]. FDA-approved JAK-STAT inhibitors are also showing promise, not only in PsA but also in different inflammatory conditions [109]. Etanercept is viewed as effective for juvenile idiopathic arthritis, which may have implications for similar strategies for psoriatic arthritis [110]. Clinical trials show sustained improvements in disease activity being achieved with Guselkumab treatment for PsA patients, indicating its potential as an effective long-term therapy option [111]. Understanding the polymorphic nature of PsA is crucial for creating individualized treatment plans [112].

In conclusion, advances in next-generation sequencing (e.g., single-cell analysis) have led to the identification of more accurate and reliable genetic markers for PsA [113,114]. There has been substantial progress in understanding the genetic underpinnings of PsA, revealing that some loci, such as TRAF3IP2, REL, and FBXL19, have a strong effect, while others, like IL23R and deletions of LCE3B and LCE3C, predispose individuals to both PsO and PsA, with certain markers, like HLA B27 at 5q31, being uniquely associated with PsA [115,116]. This enhances the potential for targeted screening within the psoriasis population to identify those at higher risk for PsA and to apply the long-needed precision medicine approach [117]. Additionally, combining genetic markers with laboratory (e.g., inflammatory markers of bone metabolism) and clinical markers (such as comorbidities and lifestyle factors) is crucial to providing targeted therapies [118]. This ongoing research continues to inform the EULAR recommendations for managing PsA with pharmacological therapies, which was last updated in 2019 [119]. Future gene function studies could provide deeper insights into disease pathogenesis, improving early diagnosis and treatment options.

## Figures and Tables

**Table 1:** PSORS with cytogenetic localizations and associated genes.

Locus Name	Cytogenetic Localization	Genes, Their Products, and Their Roles
PSORS1	6p21.3	Human leucocyte antigens (HLAs), especially HLACw6 [15]. Antigen presentation, strong association with PsO.
PSORS2	17q24-25	Missense mutation of CARD14 in keratinocytes leads to overexpression of NFκB, IL8, chemokine ligand 20, IL36, and ILγ [18]. Skin barrier functions and cellular signaling pathways.
PSORS3	4q34	Immunologically important proteins, including IL15 [15]. Influencing the inflammatory response and skin cell proliferation.
PSORS4	1q21	S100 calcium-binding proteins are overexpressed in keratinocytes of psoriatic patients and are responsible for chemotaxis of leucocytes [15]. Epidermal differentiation and barrier formation.
PSORS5	3q21	Cystatin A and zinc finger protein 148 [19]. Regulation of the immune system and inflammatory processes.
PSORS6	19p13-q13	JUNB gene produces one member of AP-1-family transcription factors controlling differentiation of keratinocytes, the KIR gene product associated with HLA antigens [20]. T-cell activation and immune response modulation.
PSORS7	1p35-p34	Gene EPS 15 codes intracellular substrate for EGF receptor, which is highly expressed in psoriatic skin [21]. Immune system and skin integrity.
PSORS8	16q	The NOD2/CARD15 gene is associated with psoriasis and Crohn’s disease [22]. Cellular proliferation, apoptosis, and the body’s inflammatory responses.
PSORS9	4q31	Polymorphism of the IL-15 gene is connected with interleukin production and inflammation [23]. Inflammation and the immune system.
PSORS10	18p11.23	EMILIN2 gene regulates apoptosis and survival of epidermal keratinocytes [24]. Cytokine production and immune regulation.
PSORS11	5q31.1-q33.1	IL-12B affects the balance of Th1/Th2 cells [25], SLC22A4,5 organic cation transporters [26], IL-13-regulating T-cell-mediated immunity [27], and IL-4,5 as Th2 cell products [28]. Immune system signaling.
PSORS12	20q13	RNF114 ring finger protein is a positive regulator of T-cell activation [29]. Inflammation and immune system responses.

**Table 2:** HLA genetic markers with supporting evidence of their association with PsA.

Marker	Evidence	Association	Study
HLA-B*08	Conflicting	-	[72,73,74,75,76]
HLA-B*13	Conflicting	-	[72,77,78]
HLA-B*18	Conflicting	-	[74,75,76]
HLA-B*27 (EH27.1 and 2)	Moderate	Positive	
HLA-B*27	Moderate	Positive	[72,73,74,75,76,78,79]
HLA-B*37	Conflicting	-	[72,76]
HLA-B*38	Conflicting	-	[74,75,76,77,78]
HLA-B*38 (EHB38.1)	Moderate	Negative	[74,75,76]
HLA-B*39	Conflicting	-	
HLA-B*57 (EH57.1)	Moderate	Negative	
HLA-B*57	Moderate	No association	[72,74,75,78]
HLA-C*01	Moderate	No association	[74,75,76]
HLA-C*02	Conflicting	-	[72,74,75,76]
HLA-C*06	Moderate	Negative	[72,73,78,79,80,81,82]
HLA-C*07	Conflicting	-	[72,74,75]
HLA-C*12	Conflicting	-	[74,75]
HLA-DRB1*03	Moderate	No association	[72,77]
HLA-DQB1*02	Conflicting	-	
HLA-B Glu45	Conflicting	-	[73,83]

**Table 3:** Non-HLA genetic markers with supporting evidence of their association with PsA.

Marker	Evidence	Association	Study
GJB2 SNP rs3751385	Moderate	Positive	[92]
ERAP1 SNP rs27524	Moderate	Positive	
IL1RN	Moderate	No association	[67,93]
IL12B SNP rs2082412	Moderate	Positive	[92]
IL12B rs3212227	Moderate	No association	[80,94]
IL12B rs6887695	Moderate	No association	
IL12B SNP rs7709212	Moderate	Positive	[23]
IL13rs1800925	Moderate	Positive	[95,96]
IL13 rs20541	Conflicting	-	[67,95]
IL23A SNP rs2066807	Moderate	No association	[67,93]
IL23R SNP rs2201841	Conflicting	-	
LCE SNP rs1886734	Moderate	Positive	[92]
PTTI SNP rs2451697	Moderate	Positive	
RUNX3 SNP rs7536201	Moderate	Positive	[23]
TNFa-238	Moderate	No association	[80,97]
TNFa-308	Moderate	No association	
TNF alpha-induced protein 3 rs610604	Moderate	No association	[98]
TNIP1 SNP rs17728338	Moderate	Positive	[92]

## Data Availability

Not applicable.
